# A novel homozygous splice variant in *DNAAF4* is associated with asthenozoospermia

**DOI:** 10.3724/abbs.2023147

**Published:** 2023-09-07

**Authors:** Xiaobin Zhu, Chen Chen, Jian Song, Shijue Dong, Xuhui Zeng, Zhihong Niu, Yanwei Sha, Xiaoning Zhang

**Affiliations:** 1 Department of Gynecology and Obstetrics Reproductive Medical Center Shanghai Ruijin Hospital Shanghai Jiao Tong University School of Medicine Shanghai 200025 China; 2 Institute of Reproductive Medicine School of Medicine Nantong University Nantong 226019 China; 3Department of Andrology Women and Children’s Hospital School of Medicine Xiamen University Xiamen 361005 China

Primary ciliary dyskinesia (PCD) is an autosomal recessive disorder with a high degree of genetic and phenotypic heterogeneity resulted from defects in the structural characteristics and motility of flagella and cilia [1]. To data, over 50 mutations have been identified as harboring PCD-related mutations, and approximately half of the affected patients suffer from infertility as a result of sperm morphological or functional abnormalities [2]. Dynein axonemal assembly factor 4 ( *DNAAF4*, also known as *DYX1C1*) is a dynein axonemal assembly family gene encoding a protein that contains a tetratricopeptide repeat domain. *DNAAF4* dysfunction was first noted in a Finnish family in which a translocation coincidentally segregating with dyslexia was noted [3]. *DNAAF4* has been found to play a role in the assembly of cilia, and mutations in this gene cause PCD as a consequence of abnormal ciliary motility and improper axonemal dynein assembly, potentially resulting in male factor infertility [4]. Very recently, it was reported that *DNAAF4* mutation is associated with asthenoteratozoospermia [2]. However, most of the reported disease-causing variants in these studies were located in the coding regions of *DNAAF4*. In this study, we provide novel evidence of an association between a newly identified *DNAAF4* homozygous splice variant and asthenozoospermia.

This study was performed under appropriate ethical review with the written informed consent of participating subjects. A 34-year-old man (P1) and his 37-year-old brother (P2) suffered from infertility for 3 and 5 years, respectively. Both completed standardized physical, clinical, and laboratory examinations, and details regarding their marriage, physical information (height and body weight), chromosome abnormality, and hormone levels were recorded ( Table 1). Ultrasound examination did not reveal any abnormalities in the seminal vesicles, prostates, bilateral testicles, epididymis, or spermatic vein in any of these patients. The peripheral blood chromosome karyotype for both patients was 46, XY, and bilateral testicular size measurements were within the normal range. There was no evidence of Y chromosome microdeletions. The wives of both patients exhibited apparently normal hormone levels and other reproduction-related tests. P1 and P2 were the offspring of a consanguineous marriage bearing four sons, of whom two were fertile.

**Table:** **Table 1** Physiological characteristics, serum indices, and semen parameters for two patients with PCD harboring *DNAAF4* mutations

Character	Patient-P1	Patient-P2	Reference limits
Age at last visit (year)	34	37	‒
Infertility duration	3	5	‒
Height (cm) at last visit	168	172	‒
Weight (kg) at last visit	61	74	‒
FSH (mIU/mL)	6.21	4.72	0.95‒11.95
LH (mIU/mL)	5.64	4.51	1.24‒8.63
T (ng/mL)	4.02	4.35	1.75‒7.8
E2 (pg/mL)	35	28	20‒75
PRL (ng/mL)	11.23	9.53	3.46‒19.4
Genetic investigation karyotype	46, XY	46, XY	‒
Y-chromosome microdeletion	No deletion	No deletion	‒
Testicular volume (mL)	15	15	‒
Seminal plasma	Normal	Normal	‒
Sperm concentration (10 ^6^/mL)	7.25±1.45	39.45±9.25	≥15
Sperm volume (mL)	2.35±0.15	2.7±0.2	≥1.5
PR (%)	0	0	≥32
PR+NP (%)	0	0	≥40
Immotile (%)	100	100	‒
Normal morphology (%)	4	6	≥4.0

FSH, follicle-stimulating hormone; LH, luteinizing hormone; T, testosterone; E2, estradiol; PRL, prolactin. Reference limits are those defined by the World Health Organization (WHO, fifth edition).

P1 exhibited obvious symptoms of PCD, including a chronic cough and recurrent airway infections. High-resolution computed tomography (CT) revealed pulmonary disease, including bronchiectasis ( Figure 1A) and situs inversus ( Figure 1B,C). Unlike his brother, P2 did not exhibit comparable PCD phenotypes, but exhibited only flagellar malfunction.

**Figure 1:**
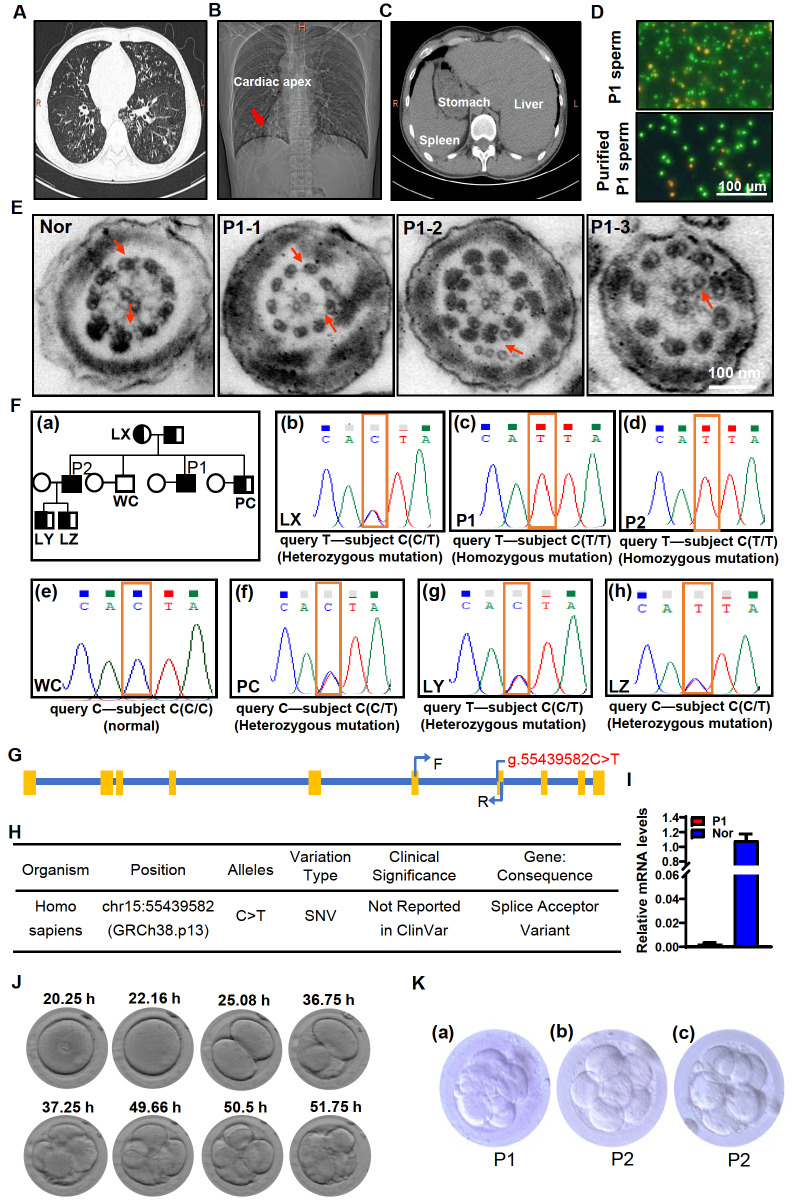
Figure 1 Investigations of chest radiography, sperm survival and flagellar ultrastructure, *DNAAF4* mutation and embryonic development X-ray and CT images from P1 showing evidence of chronic pulmonary disease with bronchiectasis (A), dextrocardia (B), and situs inversus totalis (C). (D) Survival rates for sperm from P1. A cell-permeable nucleic acid stain (green) was used to identify live sperm with membranes that were intact, while propidium iodide (orange) was used to detect dead sperm. (E) Ultrastructural images of the sperm flagella from P1 and a normal (Nor) control sample. (P1-1) Loss of the inner and outer dynein arms (IDA and ODA). (P1-2 and P1-3) Centrosome duplication and disorganization. (F) Pedigrees for the identified family impacted by the DNAAF4 mutation. The “”and “”, respectively refer to heterozygous males and females, while “” denotes homozygous males (a). DNAAF4 mutation sites for the indicated family members (b‒h). (G) DNAAF4 primer locations in the DNAAF4 mRNA sequence. (H) Specific details regarding the DNAAF4 variant recorded in dbSNP. (I) DNAAF4 mRNA levels from the P1 and normal control samples. (J) An overview of the process of normal embryonic development for the couple including P1. (K) An embryo from P1 on day 3 (a). Two embryos from P2 on day 3 (b,c).

P1 did not produce any motile spermatozoa and few (<4%) morphologically normal spermatozoa. P2 did not produce any motile spermatozoa but did exhibit a higher percentage of morphologically normal sperm (~6%). Semen analyses for both patients revealed normal volume and pH values. The sperm concentration for P1 was below the reference value (WHO, fifth edition), while that for P2 was normal ( Table 1).

Considering that no motile sperm were detected, sperm survival rates were further analyzed in detail. The survival rate of sperm from P1 was ~70% ( Figure 1D), and after purification using two-layer discontinuous density gradients (40% and 80% Percoll) [5], surviving spermatozoa accounted for 92.5% of cells ( Figure 1D). These results suggested that most of the immotile sperm in these patients were alive such that assisted reproductive technologies may be able to address the infertility affecting these patients.

In contrast to the typical axonemes exhibiting a 9+2 microtubule arrangement, as evident in sperm from a fertile control donor sample, axonemal structures in sperm from P1 and P2 exhibited pronounced ultrastructural defects. These included axonemal disorganization, inner and outer dynein arm (IDA and ODA) defects, and irregular reduplicative centrosome arrangements ( Figure 1E). These abnormalities have not been reported previously, indicating that other factors are involved in the onset of asthenozoospermia in these patients or that the novel variant identified herein exhibits effects distinct from those associated with other variants. The DNAAF4 interactome is enriched for centrosomal proteins, including CEP170, CENPJ, and NPM1, and when overexpressed, DNAAF4 reportedly exhibits centrosomal localization [6]. We therefore speculated that *DNAAF4* mutations may interfere with the normal function of the DNAAF4 complex or interacting proteins, thereby disrupting normal sperm flagellar development [ 7‒ 9].

Through whole exome and Sanger sequencing, a previously uncharacterized homozygous splice variant was identified in this gene (NC_000015.10:g.55439582C>T), altering a *DNAAF4* exon 7 consensus splice acceptor site ( Figure 1F,G). This splice variant has been recorded in dbSNP ( Figure 1H) but has not been reported in a clinical setting to date. To test whether this *DNAAF4* splice acceptor site mutation impacted the mRNA expression level of this gene, we developed a pair of primers spanning exon 6 to exon 7 ( Figure 1G). The results revealed the absence of any exon 7-containing *DNAAF4* transcripts in the sperm from P1, unlike in a normal control sample ( Figure 1I), consistent with the dysfunction or loss of DNAAF4 protein expression that may explain the abnormal sperm phenotypes in this patient.

In an effort to restore fertility for P1, intracytoplasmic sperm injection (ICSI) was performed. After the microinjection of sperm from P1 into oocytes, an 8-cell embryo scoring 7 per the evaluation criteria used by our center ( Figure 1J,K) was transferred, although a pregnancy did not successfully develop. Three additional cycles of ICSI treatment were performed for P1 over the following three years, and all embryos successfully developed to the 8-cell stage but culminated in pregnancy failure. This same approach was also employed for the couple, including P2, but they gave birth to healthy twins. Other studies have also reported similar outcomes for PCD patients [10]. These results thus suggest that a mutation located at the same position in a given gene may ultimately give rise to varying clinical symptoms. Sterile patients with severe PCD phenotypes might have a poor ICSI outcome.

In summary, we found a novel homozygous splice variant (NC_000015.10: g.55439582C>T) located in the tetratricopeptide repeat domain of the *DNAAF4* gene, altering a splice acceptor site of *DNAAF4* exon 7 and eventually lacking the expression of intact *DNAAF4* mRNA. We further verifiably linked this variation to asthenozoospermia, expanding the genetic spectrum of PCD. ICSI after sperm selection is an available treatment strategy for male infertility caused by *DNAAF4* mutation.

## Supporting information

224Supplementary_File-revised
