# Supplementary material for: A novel homozygous splice variant in DNAAF4 is associated with asthenozoospermia : DNAAF4 is associated with asthenozoospermia
Source: Acta Biochim Biophys Sin (Shanghai). 2023 Sep 7;55(10):1689–92. doi: 10.3724/abbs.2023147 (PMC10577477; doi:10.3724/abbs.2023147)
Supplement: 224Supplementary_File-revised [file 224Supplementary_File-revised.pdf]

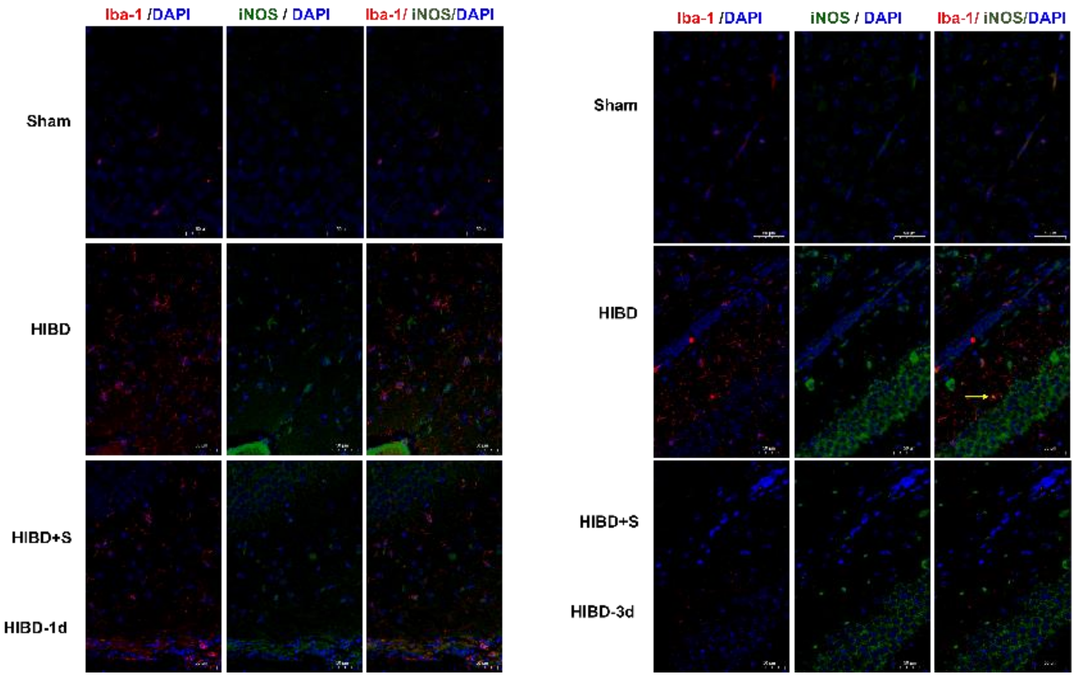

Supplementary Figure S1. The double-label immunofluorescence assay results of iNOS in HIBD mice at Day 1 and Day 3 iNOS immunofluorescence-green. Iba-1 positive microglia-red. DAPI-blue. Scale bar: 50 μm.

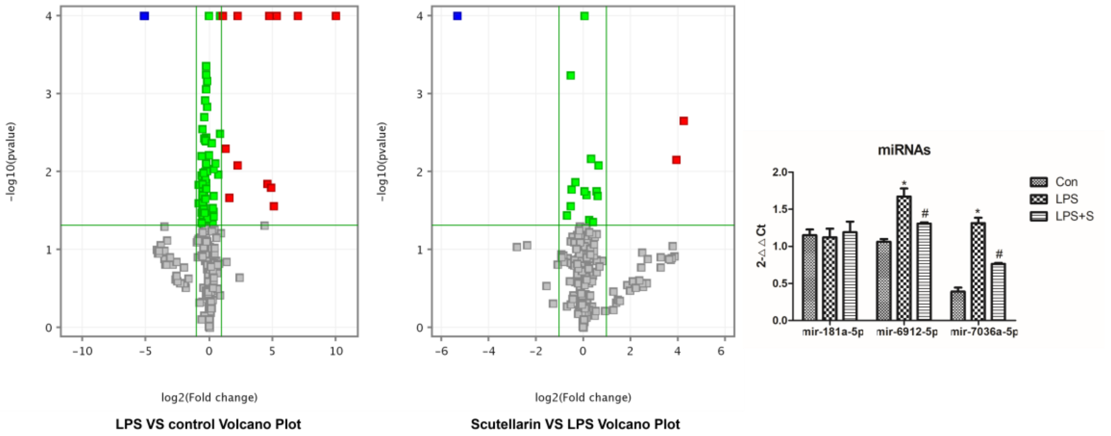

Supplementary Figure S2. The screening of miRNAs arrays The results of miRNA chip showed that a total of 14 differentially expressed miRNAs in LPS stimulated microglia compared with the control group. Meanwhile, differentially expressed miRNAs (miR-181a, miR-6912 and miR-7036a) were identified in LPS-stimulated microglia given scutellarin pretreatment. qRT-PCR showed that the expression of miR-7036a was consistent with miRNA chip results.

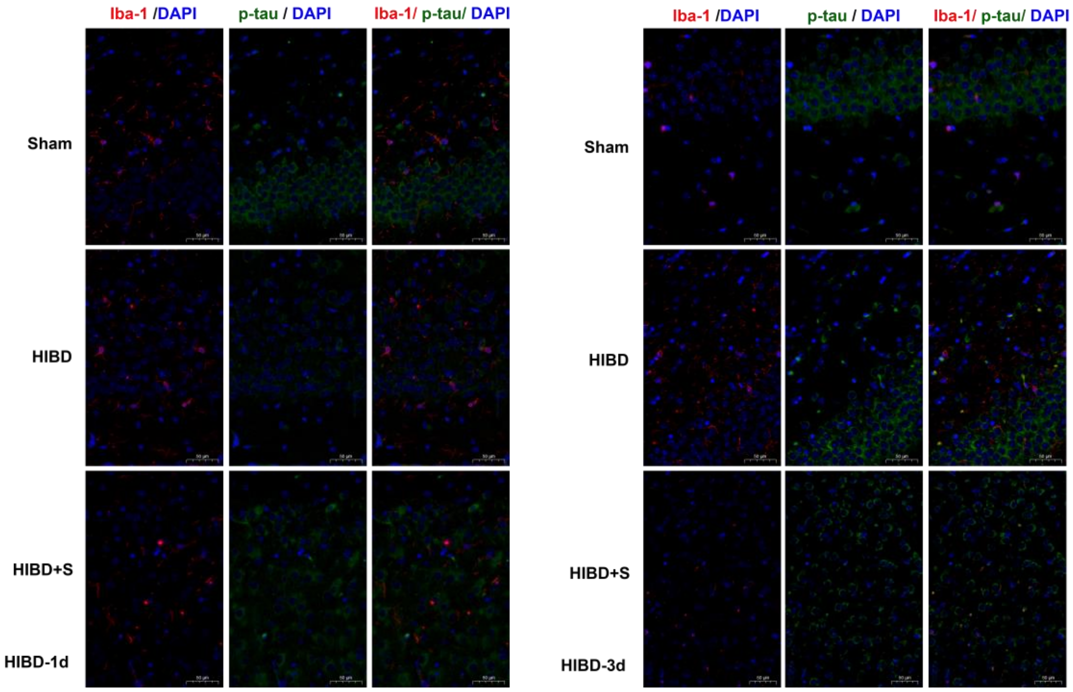

Supplementary Figure S3. The results of double-label immunofluorescence assay of p-tau in HIBD mice at Day 1 and Day 3 p-tau immunofluorescence-green. Iba-1 positive microglia-red. DAPI-blue. Scale bar: 50 μm.
